# Case Report: Severe Hypocalcemic Episodes Due to Autoimmune Enteropathy

**DOI:** 10.3389/fendo.2021.645279

**Published:** 2021-06-14

**Authors:** Inbal Halabi, Marie Noufi Barohom, Sarit Peleg, Phillippe Trougouboff, Ghadir Elias-Assad, Rhania Agbaria, Yardena Tenenbaum-Rakover

**Affiliations:** ^1^ Pediatric Endocrine Institute, Ha’Emek Medical Center, Afula, Israel; ^2^ Pediatric Health Center, Clalit Health Services, Naharia, Israel; ^3^ Faculty of Medicine, Bar Ilan University, Safed, Israel; ^4^ Pediatric Health Center, Clalit Health Services, Hadera, Israel; ^5^ Tissue Diagnosis and Cancer Research Department, Ha’Emek Medical Center, Afula, Israel; ^6^ The Ruth & Bruce Rappaport Faculty of Medicine, Technion, Haifa, Israel; ^7^ Pediatric Gastroenterology Unit, Ha’Emek Medical Center, Afula, Israel

**Keywords:** autoimmune polyglandular type 1 syndrome, autoimmune polyendocrinopathy-candidiasis-ectodermal dystrophy, autoimmune regulator gene, autoimmune enteropathy, hypocalcemia, enteroendocrine cell, chromogranin A

## Abstract

Autoimmune polyendocrinopathy-candidiasis-ectodermal dystrophy (APECED) is a rare monogenic disorder, associated with endocrine deficiencies and non-endocrine involvement. Gastrointestinal (GI) manifestations appear in approximately 25% of patients and are the presenting symptom in about 10% of them. Limited awareness among pediatricians of autoimmune enteropathy (AIE) caused by destruction of the gut endocrine cells in APECED patients delays diagnosis and appropriate therapy. We describe an 18-year-old female presenting at the age of 6.10 years with hypoparathyroidism, oral candidiasis and vitiligo. The clinical diagnosis of APECED was confirmed by sequencing the autoimmune regulator-encoding (*AIRE*) gene. Several characteristics of the disease—Hashimoto’s thyroiditis, Addison’s disease, diabetes mellitus type 1 and primary ovarian insufficiency—developed over the years. She had recurrent episodes of severe intractable hypocalcemia. Extensive GI investigations for possible malabsorption, including laboratory analyses, imaging and endoscopy with biopsies were unremarkable. Revision of the biopsies and chromogranin A (CgA) immunostaining demonstrated complete loss of enteroendocrine cells in the duodenum and small intestine, confirming the diagnosis of AIE. Management of hypocalcemia was challenging. Only intravenous calcitriol maintained calcium in the normal range. Between hypocalcemic episodes, the proband maintained normal calcium levels, suggesting a fluctuating disease course. Repeated intestinal biopsy revealed positive intestinal CgA immunostaining. The attribution of severe hypocalcemic episodes to AIE emphasizes the need for increased awareness of this unique presentation of APECED. The fluctuating disease course and repeated intestinal biopsy showing positive CgA immunostaining support a reversible effect of GI involvement. CgA immunostaining is indicated in patients with APECED for whom all other investigations have failed to reveal an explanation for the malabsorption.

## Introduction

Autoimmune polyendocrinopathy-candidiasis-ectodermal dystrophy (APECED), also known as autoimmune polyendocrine syndrome type 1, is a rare monogenic disorder caused by mutations in the autoimmune regulator gene (*AIRE*; OMIM 240300). *AIRE* plays a critical role in central immune tolerance (1, 2). The classic triad manifestations of APECED are chronic mucocutaneous candidiasis, hypoparathyroidism and adrenal insufficiency. Developing any two of these three major components of the disease establishes the diagnosis clinically. Multiple organs with autoimmune involvement have been reported in patients with APECED (3–8).

Gastrointestinal (GI) manifestations appear in approximately 25% of APECED patients and in about 10%, they are the presenting symptom. GI symptoms include malabsorption, constipation, watery diarrhea or steatorrhea (9). Autoimmune enteropathy (AIE) as part of APECED was first described in 1982 (9) but since then, only small series and case reports have been published (10–12). The mechanism leading to GI involvement in AIE is still not completely understood. Reduction or absence of enteroendocrine (EE) cells has been shown to be the main specific contributor to intestinal dysfunction (10–12).

The EE cells are scattered throughout the GI tract and constitute the gut endocrine system. Different EE cell types produce different gut hormones. These cells have important roles in gut growth, blood flow, motility, and secretion of pancreatic enzymes, bile and bicarbonate-rich fluid (13–15). The most common type of EE cell is the enterochromaffin cell, which secretes mainly serotonin and is dispersed throughout the GI system. Serotonin is synthesized by the hydroxylation and decarboxylation of tryptophan (14), and tryptophan hydroxylase has been identified as the intestinal autoantigen in APECED (16); moreover, anti-tryptophan hydroxylase antibodies are a highly specific marker for autoimmune GI involvement (16, 17).

Chromogranin A (CgA) colocalizes with almost all EE cell types in the different parts of the human GI tract, and it is therefore regarded as a surrogate marker for these cells (18). EE cells, which are lacking in AIE as part of APECED syndrome, are not affected in other intestinal diseases (10). Moreover, except for the absence of EE cells, intestinal histology is normal in APECED (9).

Herein, we describe an 18-year-old female diagnosed with APECED who first presented with hypoparathyroidism, vitiligo and chronic mucocutaneous candidiasis; over the years, she developed Hashimoto’s thyroiditis, diabetes mellitus type 1, Addison’s disease and primary ovarian insufficiency. The patient’s disease course, which involved recurrent admissions to the hospital with GI symptoms and severe persistent hypocalcemia that required prolonged hospitalizations, led to the diagnosis of AIE confirmed by intestinal CgA immunostaining. This report highlights AIE as one of the unrecognized factors of GI involvement in APECED and discusses the challenges of managing hypocalcemia associated with AIE.

## Case Description

**Figure 1:**
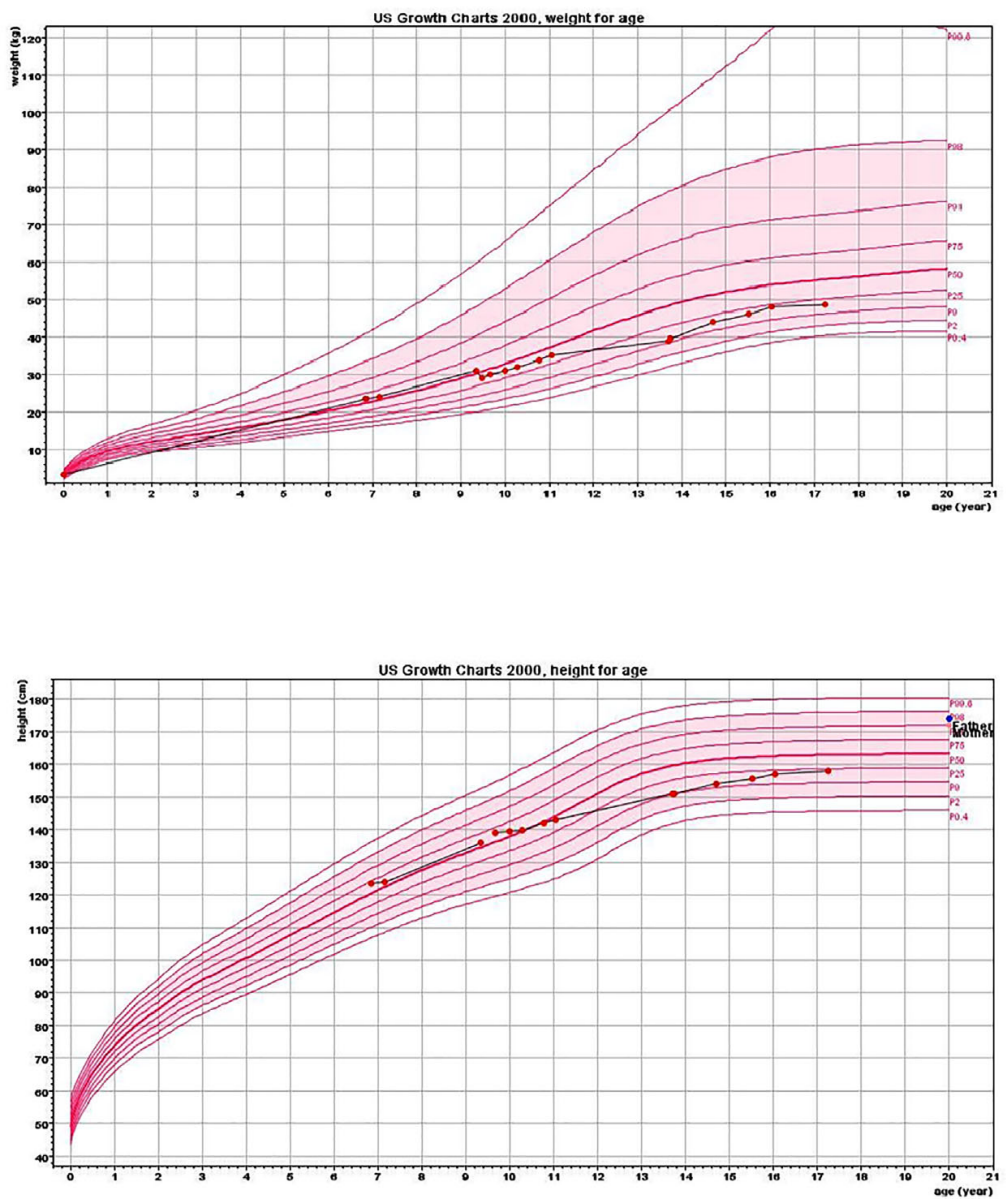
Height and weight charts. Charts show growth deceleration from the 60^th^ to 10^th^ centile and a lack of weight gain from the age of 9.5 years.

**Figure 2:**
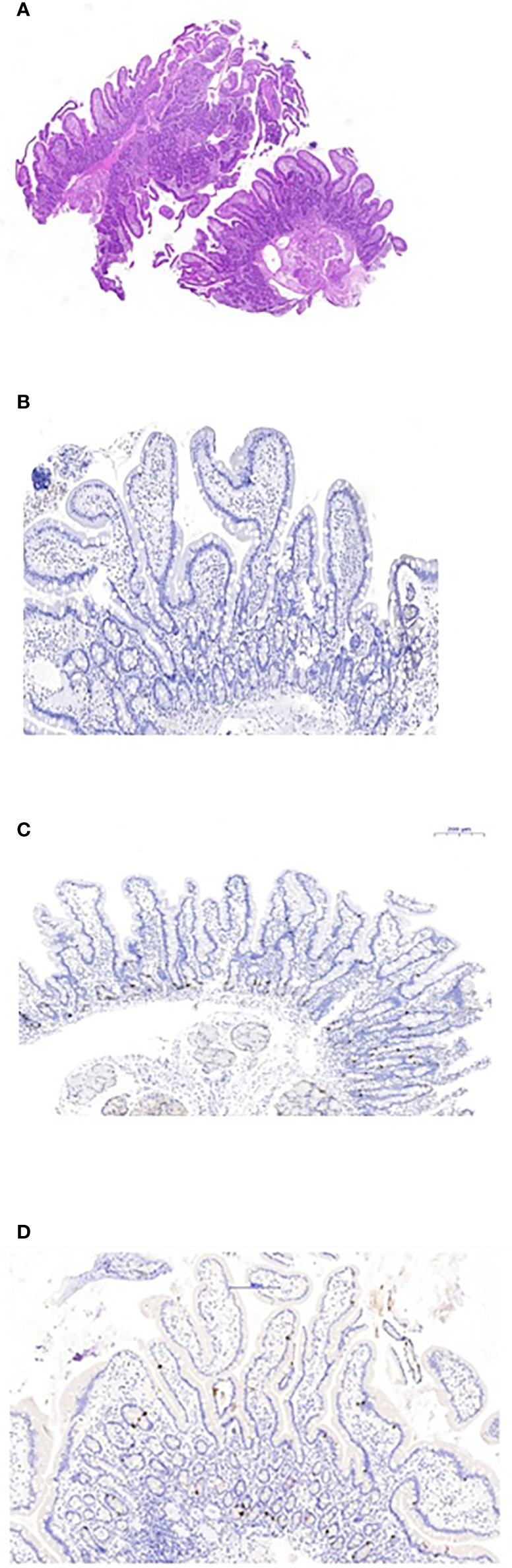
Duodenal biopsies. **(A)** Hematoxylin and eosin staining shows normal duodenal mucosa without villous atrophy. **(B)** CgA immunostaining of the duodenal biopsy shows complete loss of EE cells. **(C)** Normal control duodenal mucosa immunostained with CgA, showing EE cell distribution in the crypt epithelium. **(D)** Repeated biopsy shows normal immunostained CgA duodenal mucosa.

The proband was an 18-year-old female of Christian–Arab descent, whose parents were first-degree cousins. At the age of 6.10 years, she presented to the rheumatological clinic with episodes of muscle spasm, leg pain, recurrent fever, intermittent abdominal pain and diarrhea. Laboratory evaluation revealed no remarkable findings. Ten months later, she was admitted to our medical center due to severe hypocalcemia (4.5 mg/dL; normal range, 8.1–10.6), elevated phosphorus (9.11 mg/dL; normal range, 2.5–4.5) and undetectable serum parathyroid hormone levels (less than 1 pg/mL; normal range, 12–65). These findings were consistent with the diagnosis of acquired hypoparathyroidism. On examination, she had oral candidiasis and vitiligo on the knees, left elbow, and around the mouth and eyes. Presence of the two major components of the disease (hypoparathyroidism and chronic mucocutaneous candidiasis) and one minor component (vitiligo) resulted in a clinical diagnosis of APECED. Administration of calcium supplement (40 mg/kg elemental calcium daily) and α-hydroxycholecalciferol (alpha D3; 0.25 µg daily) was initiated and at these doses, calcium was maintained at the desired level (above 7.5 mg/dL). At the age of 9 years, positive thyroid antibodies were detected with normal thyroid function and the diagnosis of euthyroid Hashimoto’s thyroiditis was made (**Tables 1** and **2**). On follow up, the proband underwent recurrent hospitalizations in the intensive care unit due to severe hypocalcemia which was difficult to normalize. At the age of 9.6 years, she was admitted to our hospital due to fatty acholic diarrhea, abdominal pain and a fever lasting 10 days. She had not gained weight for 12 months prior to her admission (**Figure 1**). Laboratory evaluation revealed severe hypocalcemia. Despite high doses of intravenously (IV) administered calcium (180 mg/kg elemental calcium daily) along with oral calcium supplements of 56 mg/kg elemental calcium per day, alpha D3 (5 µg daily; 0.17 µg/kg daily) and a magnesium supplement, calcium levels remained low. It was only after initiation of calcitriol, administered IV at a dose of 0.5 µg three times a week that calcium levels were maintained. Calcitriol (1 microgram/ml) Kern Pharma, S.L.Spain. N° Reg.: 68.934. This hospitalization lasted 1 month. The episodes of hypocalcemia that required hospitalization were preceded by GI manifestations, including abdominal pain, bloating, diarrhea, constipation and fatty stool, and therefore an extensive GI investigation was carried out with unremarkable findings. Imaging of the upper GI tract, including a radiological GI series, demonstrated normal gastric emptying and normal bowel motility without any pathological findings for inflammatory bowel disease. Ultrasonographic scans of the abdomen were without liver or pancreatic anomalies. Upper GI endoscopic biopsies showed mild lymphocytic esophagitis, and the gastric biopsy demonstrated a picture of autoimmune atrophic gastritis with reduction in acid-secreting parietal cells, without intestinal metaplasia. There was no evidence of *Helicobacter pylori* and no villous atrophy of the duodenal or terminal ileal mucosa, and the number of intraepithelial lymphocytes was within the normal range. No active inflammation or granulomas were seen in the biopsies of the terminal ileum or colonic mucosa. Colonoscopy with biopsies and magnetic resonance elastography of the bowel excluded inflammatory bowel disease. Liver needle biopsy revealed no significant histopathological changes. Following these extensive GI investigations, which excluded exocrine pancreas insufficiency, inflammatory bowel disease, celiac disease, GI infections and autoimmune hepatitis, the diagnosis of AIE was raised, and specific CgA immunostaining of the stomach, duodenal and ileal biopsies was performed. This revealed a complete absence of EE cells in the duodenum and the small intestine with almost normal staining of the stomach, confirming the diagnosis of AIE (**Figure 2**). After this long hospitalization, during the follow-up period, she maintained calcium serum levels within 7.5 to 10.0 mg/dL with some fluctuations, with calcium supplement of around 20 mg/kg/d and α-D3 of 2 µg daily. At the age of 13.8 years, following 4 weeks of polyuria and polydipsia, blood glucose was 450 mg/dL and HbA1c was 9.1% (normal range <5.6%). The diagnosis of diabetes mellitus type 1 was made (**Table 2**), and insulin injections were initiated. Two months later, adrenocorticotropic hormone stimulation test revealed peak cortisol of 10.53 µg/dL—consistent with partial adrenal insufficiency—and oral glucocorticoid administration (10 mg/m^2^ per day, hydrocortisone) was initiated (**Table 3**). At the same age, the proband had no signs of pubertal development and growth deceleration was seen, with a decrease from the 60^th^ to 10^th^ centile for height (**Figure 1**). Elevated gonadotropins confirmed a diagnosis of primary ovarian insufficiency (**Table 3**), and supplemental estradiol treatment was initiated. At the age of 18 years, she was admitted to our Medical Center due to coffee ground vomiting, loss of appetite and weight loss. Laboratory evaluation revealed hypercalcemia of 13.11 mg/dL that was normalized by IV 0.9% NaCl solution. Repeated intestinal biopsy revealed normal numbers of positive CgA-immunostained cells in the duodenum. Stomach biopsies demonstrated a *Helicobacter pylori*-positive active gastritis and an associated lymphocytic gastritis, possibly related to *H. pylori* as well. In a biopsy from the stomach body, no cells were present with CgA immunostaining. Sequencing of *AIRE* identified a previously described homozygous missense mutation (c.47C>T, p.Thr16Met), confirming the diagnosis of APECED. She is currently 20 years old and she graduated high school with distinguish.

**Table 1:** Summary of the biochemical results and the treatment modalities over the years in the proband.

Date	4 Nov 2006	12 Nov 2008	17 Nov 2011	23 May 2011	29 May 2011	13 Sep 2011	18 Sep 011	27 Sep 2011	16 Oct 2011	31 May 2019	18 Jul 2020	28 Jan 2021	Normal ranges
Age (years)	6. 75		9.3			9.75				17.2	18.5	19	
Calcium (mg/dL)	5.27	7.9	5.71	6.64	7.41	6.67	5.65	7.91	8.71	6.67	14.24	8.4	8.5–10.5
Phosphorous (mg/dL)	12.21	8.96	7.94	8.2	6.8	4.86	7.88	5.59	5.87	5.08	3.07	5.4	2.5–5.0
Vitamin D (25-OH) (ng/mL)	10.6	ND	ND	ND	ND	ND	8.3	ND	76	<10.5	36.7	ND	30–100
Magnesium (ng/mL|)	1.79	1.96	1.44	1.68	1.66	1.54	1.68	1.79	1.52	1.34	2.4	1.9	1.7–2.55
**Management**													
Oral calcium supplements (elemental calcium mg/kg/day)	_	68	64.5	129	129	129	129	56	56	30	51	10	
Type of calcium supplement		Calcium Sandoz	Calcium Sandoz	Calcium Sandoz	Calcium Sandoz	Calcium Sandoz	Calcium Sandoz	Calcium carbonate	Calcium carbonate	Calcium carbonate	Calcium carbonate	Calcium carbonate	
IV calcium gluconate (mg/kg/d)	_	Yes	_	Yes	_	90	180	No	_	Yes			
α- D3 (µg/d)	_	0.5	0.75	2	3	5	5	4	5	4	4	2	
IV calcitriol (µg every 2 days)	_	_	_	_	_	_	_	0.5 x 10 doses	_	_	_	_	
Magnesium (mg/d)	_	No	No	No	No	100	100	100	100	520	1040	1560	
Symptoms	Muscle spasm, leg pain		Acholic diarrhea			Fever, diarrhea, weight loss				Vomiting, abdominal pains	Hematemesis, weight loss, abdominal pains		
Comments	At diagnosis (ICU)	Discharge	Admission to pediatric department		Discharge	Admission to pediatric department	At hospital	At hospital	Discharge	ICU	ICU	Last visit	

ND, not done.

**Table 2:** Disease manifestation, age at onset and treatment.

Age (years)	Manifestation	Antibody	Result	Treatment	Normal range
4.8	Vitiligo				
4.8	Mucocutaneous candidiasis			Ketoconazole orally	
6.8	Hypoparathyroidism			Calcium supplement 40 mg/kg per day Alpha D3 0.25 µg/day	
7.9	Partial adrenal insufficiency			Hydrocortisone in stress	
9	Hashimoto’s thyroiditis	TPO Ab	208		0-35
		TG Ab	185		0-35
9.9	Autoimmune enteropathy				
9.9	Nail dystrophy				
13.8	Diabetes Mellitus type 1	Anti-GAD	7.1	Insulin (0.8 units/day)	<1.0 U/mL
		IA2	6.6		<0.75 U/mL
		Anti-insulin	<7.0		<7%
13.10	Primary ovarian insufficiency			Estradiol	
13.10	Adrenal insufficiency	Anti-adrenal	Positive	Hydrocortisone 10 mg/m^2^ per day	Negative
18	Atrophic gastritis	Intrinsic-factor	130		0-20
		Anti-parietal	Positive		Negative

**Table 3:** Hormonal results.

	Age (years)	6.1	7.11	9.4	13.10	17	Normal range
	FT_4_ (pmol/L)	18.3		15.1	16.4	14.9	9.9-22.7
	TSH (mIU/L)	3.3		2.3	2.44	7.37	0.4-4.2
	Prolactin (ng/dL)			6.1	6.22		2.1-17.7
	IGF-I (ng/mL)			157	225.7		116-358^α^
	IGFBP-3 (ng/mL)			3570		4670	3400-9500^α^
	Basal GH (ng/mL)			4.9		22.8	>5.0
	Estradiol (pg/mL)			14.1		13.7	
GnRH	Basal LH (mIU/mL)			1.4	28.6		1.1-7.4^α^
	Peak LH (mIU/mL)				139.2		10.4-34.4^α^
	Basal FSH (mIU/mL)			12.2	>200		0.3-4.8^α^
	Peak FSH (mIU/mL)				>200		12.2-19.9^α^
ACTH	Basal cortisol (µg/dL)	13.2	5.3	16.5	6.9		>10
	Peak cortisol (µg/dL)	21.8	16.7	21.2	10.5		>20
	PTH (pg/mL)	<1.0		<0.3			12.0-65.0 pg/mL

FT_4_, free thyroxine; TSH, thyroid-stimulating hormone; IGF-1, insulin-like growth factor 1; IGFBP-3, IGF binding protein-3; GnRH, gonadotropin-releasing hormone; ACTH, adrenocorticotropic hormone; PTH, parathyroid hormone.

^α^Normal ranges for adult.

## Discussion

We describe a girl with APECED who first presented with hypoparathyroidism, and then developed several characteristics of the disease over the years. Intestinal involvement due to AIE caused recurrent episodes of severe, difficult to manage hypocalcemia. There is limited awareness of GI involvement in APECED (3, 9). Our knowledge of AIE as part of APECED was gathered from small series and case reports (9–12, 19). The precise mechanism of autoimmune intestinal involvement is not well understood. More than 90% of patients with intestinal dysfunction have been found positive for anti-tryptophan hydroxylase antibodies (20).

We demonstrated a total loss of EE cells in the duodenal and small bowel mucosa by lack of CgA staining in intestinal biopsies. The recurrent episodes of persistent, severe hypocalcemia following the GI manifestations suggested that the hypocalcemic episodes are a consequence of intestinal malabsorption. However, extensive malabsorption workup excluded exocrine pancreatic insufficiency as a cause. The pathophysiology of the EE cell loss’s interference with calcium and/or vitamin D absorption is unclear. Infants with *NEUROG3* mutations, which result in congenital dysgenesis of EE cells, present with severe, life-threatening watery diarrhea (21). The absence of EE cells in this congenital disease emphasizes their important role in intestinal absorption (10). An animal model suggests that *Neurog3* is an important transcription factor in the development and differentiation of EE cells (15). The small intestine, and mainly the duodenum, are primarily responsible for calcium absorption, through active transport stimulated by 1,25-dihydroxyvitamin D (22). EE cells have been recently shown to play an important role in the digestion of macronutrients into their component parts: glucose, amino acids, and fatty acids. These small molecules are then detected by specific transporters and receptors located on the EE cells, and stimulate hormone secretion predominantly at the sites where nutrient absorption is maximal. It seems that malabsorptive diarrhea results from the loss of EE cells (15) and hypocalcemic episodes resulted from malabsorption of oral calcium and vitamin D. Moreover, in each such hypocalcemic episode, a stress dose of hydrocortisone was given due to adrenal insufficiency, which might reduce intestinal calcium absorption, renal calcium resorption, and bone remodeling, further decreasing the proband’s serum calcium levels (23). Furthermore, hypocalcemia itself can cause malabsorption by reducing cholecystokinin secretion, decreasing gall bladder emptying, and causing pancreatic insufficiency (7, 23). The association between loss of EE cells and diarrhea in patients with APECED and severe hypocalcemia that is unresponsive to conventional therapy has been reported by others as well (19, 23–25).

Management of hypocalcemia in such cases is extremely challenging. Despite a high dose of calcium supplements and alpha D3, calcium levels remained very low in the proband. Only IV calcitriol maintained calcium in the normal range. It has been shown that parenteral vitamin D analogs are superior to oral calcitriol for the long-term control of hyperparathyroidism in dialysis patients (26). However, no data has been reported on the efficacy of IV calcitriol in patients with hypocalcemia related to other conditions. Our experience indicates that IV calcitriol should be consider in unresponsive hypocalcemia related to AIE.

At present, there are no clear guidelines for the treatment of hypoparathyroidism in children and adolescents in general, and even less so in hypoparathyroidism associated with AIE (27).

Immunosuppressive therapy in a patient with APECED presenting with unresponsive severe hypocalcemia led to improved calcium absorption and maintenance of normal calcium levels (25). However, this improvement could also be attributed to the fluctuating course of AIE, and be independent of the effect of the immunosuppressive therapy; further studies are therefore needed to justify the benefit of such aggressive therapy (10). Interestingly, our patient demonstrated a fluctuating disease course and fluctuating serum calcium levels, suggesting reversible involvement of EE cells. Indeed, in contrast to the first biopsy, repeated intestinal biopsy revealed normal CgA immunostaining in the duodenum, indicating a regenerative process; however, no CgA staining was demonstrated in the stomach. This discrepancy may attributed to different regeneration time between the stomach and the duodenum as was demonstrated in mice model (28). Contrary to this concept, Barrett et al., have shown that endocrine cells of the gut in human have no proliferative capacity (29). This seems to contrast with the classic endocrine manifestations of APECED, such as hypoparathyroidism, Addison’s disease and primary ovarian insufficiency, which are permanent (3, 19).

Recently, treatment involving injections of recombinant parathyroid hormone has been recommended in hypoparathyroidism that does not respond to conventional therapy. This treatment is not yet approved in children, and the possible risk of osteosarcoma in childhood has to be taken into consideration (30).

Finally, sequencing of *AIRE* revealed a homozygous missense mutation (c.47C>T, p.Thr16Met) that has been previously described in patients of Russian (31) and Greek (32) origin, and is predicted to be pathogenic according to bioinformatics tools. This is a common mutation in the Israeli Christian–Arab population, known as a founder mutation.

## Conclusions

We report on a patient with APECED in which severe episodes of hypocalcemia were related to calcium and vitamin D malabsorption due to AIE, and discuss the challenging management of this presentation. CgA staining is indicated in patients with APECED and GI involvement when all other investigations fail to reveal an explanation for the malabsorption. This report highlights the need for more awareness among pediatricians of the entity of AIE as an important clinical presentation in APECED.

## Data Availability Statement

The original contributions presented in the study are included in the article/supplementary material. Further inquiries can be directed to the corresponding author.

## Ethics Statement

Written informed consent was obtained from the individual(s), and minor(s)’ legal guardian/next of kin, for the publication of any potentially identifiable images or data included in this article.

## Author Contributions

All authors listed have made a substantial, direct, and intellectual contribution to the work, and approved it for publication.

## Conflict of Interest

The authors declare that the research was conducted in the absence of any commercial or financial relationships that could be construed as a potential conflict of interest.

## Abbreviations

AIE, autoimmune enteropathy; AIRE, autoimmune regulator; APECED, autoimmune polyendocrinopathy-candidiasis-ectodermal dystrophy; CgA, chromogranin A; EE, enteroendocrine; GI, gastrointestinal.
